# Systemic Acquired Resistance in Moss: Further Evidence for Conserved Defense Mechanisms in Plants

**DOI:** 10.1371/journal.pone.0101880

**Published:** 2014-07-07

**Authors:** Peter S. Winter, Collin E. Bowman, Philip J. Villani, Thomas E. Dolan, Nathanael R. Hauck

**Affiliations:** 1 Department of Pharmacology and Cancer Biology, Duke University, Durham, NC, United States of America; 2 Indiana University School of Medicine, Indiana University, Muncie, IN, United States of America; 3 Department of Biological Sciences, Butler University, Indianapolis, IN, United States of America; Portland State University, United States of America

## Abstract

Vascular plants possess multiple mechanisms for defending themselves against pathogens. One well-characterized defense mechanism is systemic acquired resistance (SAR). In SAR, a plant detects the presence of a pathogen and transmits a signal throughout the plant, inducing changes in the expression of various pathogenesis-related (PR) genes. Once SAR is established, the plant is capable of mounting rapid responses to subsequent pathogen attacks. SAR has been characterized in numerous angiosperm and gymnosperm species; however, despite several pieces of evidence suggesting SAR may also exist in non-vascular plants^6–8^, its presence in non-vascular plants has not been conclusively demonstrated, in part due to the lack of an appropriate culture system. Here, we describe and use a novel culture system to demonstrate that the moss species *Amblystegium serpens* does initiate a SAR-like reaction upon inoculation with *Pythium irregulare*, a common soil-borne oomycete. Infection of *A. serpens* gametophores by *P. irregulare* is characterized by localized cytoplasmic shrinkage within 34 h and chlorosis and necrosis within 7 d of inoculation. Within 24 h of a primary inoculation (induction), moss gametophores grown in culture became highly resistant to infection following subsequent inoculation (challenge) by the same pathogen. This increased resistance was a response to the pathogen itself and not to physical wounding. Treatment with β-1,3 glucan, a structural component of oomycete cell walls, was equally effective at triggering SAR. Our results demonstrate, for the first time, that this important defense mechanism exists in a non-vascular plant, and, together with previous studies, suggest that SAR arose prior to the divergence of vascular and non-vascular plants. In addition, this novel moss – pathogen culture system will be valuable for future characterization of the mechanism of SAR in moss, which is necessary for a better understanding of the evolutionary history of SAR in plants.

## Introduction

Plants use various methods to defend themselves against pathogen attack. Cuticles and cell walls provide physical barriers to infection [1], while the production of phytoalexins and other antimicrobial compounds can directly interfere with the survival and spread of the pathogen [2]. Two inducible defense systems have been well-characterized in vascular plants: a localized hypersensitive response (HR) and plant-wide systemic acquired resistance (SAR) [3], [4]. HR is characterized by ion fluxes, the generation of reactive oxygen species, and localized programmed cell death, which are governed by interactions among the products of pathogen avirulence genes and those of plant resistance genes [3]. In SAR, a plant detects the presence of a pathogen and transmits a signal throughout the plant via the phloem, inducing changes in the expression of various pathogenesis-related (PR) genes [4], [5]. Once SAR is established, the plant is capable of mounting rapid responses to subsequent attacks from a wide range of pathogens. The plant hormones jasmonic acid (JA), salicylic acid (SA), and ethylene (ET) are shown to play roles in activating pathogenesis-related (PR) defense genes and establishing systemic resistance [5]. Specifically, pathogenesis-related (PR) genes are shown to be associated with signaling pathways and subsequent establishment of systemic resistance. These PR genes encode various antimicrobial products, including β-1,3 glucanases and chitinases [4]. SAR has been characterized in numerous angiosperms and at least one gymnosperm species [6], [7].

Recent studies of plant-pathogen interactions involving a model nonvascular plant, the moss *Physcomitrella patens*, have revealed host-plant responses similar to those seen in vascular plants [8], [9], [10], [11]. Oliver et al. showed that inoculation with the broad-spectrum fungal pathogen *Pythium irregulare* resulted in increased levels of reactive oxygen species and cell death [11]. Up-regulation of the defense related plant hormone jasmonic acid (JA) and its precursor 12-oxo phytodienoic acid (OPDA) were also noted 24 h after the initial inoculation, and callose was deposited at the sites of attempted penetration by the pathogen [11]. Ponce de Leon et al. demonstrated that whole-plant treatment with elicitors or cell-free culture filtrates of the bacterium *Erwinia carotovora* or inoculation with spores of the fungus *Botrytis cinerea* altered expression of the genes *PR-1*, *CHS*, *PAL*, and *LOX*, which are all up-regulated upon pathogen attack in vascular plants [9]. Similarly, exogenous JA application also induces *CHS*, *PAL*, and *LOX* gene expression in *P. patens* [11].

While these studies suggest the presence of conserved defense mechanisms and the possibility of a SAR mechanism in Bryophytes, direct phenotypic evidence of systemic resistance will be necessary before claims of SAR in non-vascular plants can be made. In this study, we used a different moss species, *Amblystegium serpens*, and the pathogenic oomycete *P. irregulare*. *P. irregulare* is a broad-spectrum necrotic pathogen, which grows quickly in culture and has been used in a previous moss-pathogen study [11]. *A. serpens*, the creeping feather moss, was used rather than the traditional model moss species *P. patens*, because of its more prostrate growth form, which offers the experimental advantage of separating the sites of induction and challenge inoculations by a physical distance. Thus, two discrete responses can be examined: one at the site of infection (HR response), as well as the effects of this initial infection on a distal, isolated site (SAR response). Using this novel culture system, we offer the first conclusive evidence that an SAR-like response occurs in non-vascular plants. Such direct evidence of systemic resistance is necessary to determine if this important plant defense system evolved before or after the divergence of vascular and non-vascular plants, estimated to have occurred at least 450 million years ago [12].

## Materials and Methods

### Moss and pathogen growth conditions

The moss, *A. serpens*, was collected from the Butler University campus, sterilized with a diluted bleach solution (.785% free chlorine), and grown in sterile cultures on Murashige and Skoog medium (MS medium, Caisson Laboratory, North Logan, Utah, USA). Cultures were maintained at 22°C with a photoperiod regimen of 16 h light and 8 h dark. 4-cm long moss gametophores were cut from sterile moss plants and grown in culture for 3 wks prior to experimentation. The oomycete, *P. irregulare*, was cultured on potato dextrose agar (PDA, Sigma-Aldrich, Saint Louis, Missouri, USA) and were maintained at 22°C.

### Pathogen inoculation and evaluation of *A. serpens* infection

*P. irregulare* inoculations were performed as described elsewhere with slight modifications [11]. Agar plugs (3-mm^3^) were taken from the growing front of a *P. irregulare* culture and placed directly onto one end of a 4-cm long moss gametophore. Care was taken to avoid contact between plugs of inoculum and the moss medium. PDA plugs without *P. irregulare* were used as controls. Growth of *P. irregulare* and progression of infection symptoms were observed microscopically 24 h post-inoculation by trypan blue staining (0.01% in lactophenol) [13]. Survival of *A. serpens* was observed for 10 d following inoculation.

### SAR investigation

**Figure 1:**
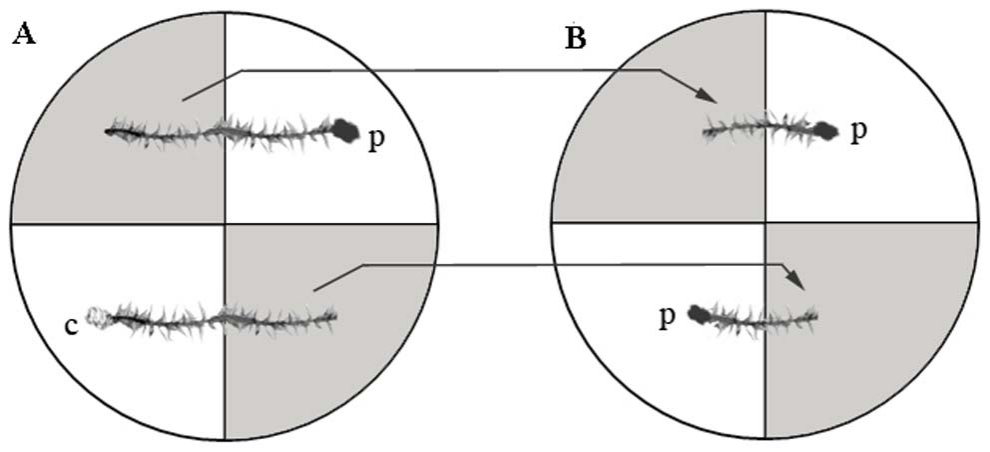
Experimental set-up for the induction and challenge experiments. Two non-adjacent sections of a four-section Petri dish were filled with MS medium (gray); the other two sections remained empty (white). (**A**) For induction inoculations, two 4-cm-long *A. serpens* gametophores were placed on the medium so that half of each sample was suspended in air above the empty Petri dish sections. PDA plugs that were approximately 3 mm^3^ in size, either containing the pathogen *P. irregulare* (p) or not (c), were placed on the suspended end of the gametophore. (**B**) After 10 h, the moss gametophores were cut in half, and the unexposed halves of the two moss gametophores were placed into a new divided plate with the distal-most ends suspended in air. New PDA plugs containing the pathogen (p) were placed on the unexposed end either 0, 12, or 24 h after the cut and transfer. One week after the challenge inoculation, samples were observed for degree of chlorosis and necrosis. p, PDA plugs with the pathogen *P. irregulare*; c, control PDA plugs without the pathogen.

SAR experiments were conducted in four-section Petri dishes. Two non-adjacent sections of a dish were filled with MS medium, and the remaining two sections were left empty (Figure 1). For induction inoculations, 4-cm-long *A. serpens* gametophores were placed in the medium so that half of each moss sample was suspended in air above the empty Petri dish sections. The suspended end of each 4-cm moss gametophore served as the site of induction inoculation. A 3-mm^3^ PDA plug containing the pathogen was gently placed on the suspended *A. serpens* tissue (Figure 1A). This ensured that *P. irregulare* would infect the moss tissue at a distinct site rather than simply growing through the medium and either by-passing the moss or initiating multiple infection sites. As a control, PDA plugs without pathogen were placed on separate *A. serpens* gametophores. Moss gametophores were cut in half 10 h after induction, and the uninoculated half was moved to a new Petri dish, now with the end furthest from the induction site suspended in air. A fresh PDA plug containing the pathogen was placed on the suspended end of this gametophore, thus constituting a challenge site (Figure 1B). Moss samples were observed for approximately 10 d after challenge for symptoms of infection. To determine the timing of systemic resistance induction, challenge inoculations were performed at 10, 22, and 34 h after induction inoculation (*i.e*., 0, 12, and 24 h after cutting), and the health of the plants was observed as described above. Four replicates, containing four gametophores each, were performed for each treatment, representing a total of 16 moss samples. The percent survival was recorded for each of the four replicates for each treatment.

### PCR amplification of *P. irregulare* ribosomal ITS1 region

We used PCR to confirm that the distal ends of inoculated *A. serpens* gametophores were not directly exposed to *P. irregulare*. Ten *A. serpens* gametophores were inoculated with a *P. irregulare* plug. Ten hours after induction inoculation, the *A. serpens* gametophores were cut in half and the two ends of each gametophore (proximal to the site of inoculation and distal to the inoculation) were moved to separate media for an additional 24 h to detect growth of existing *P. irregulare*. DNA was then extracted from each half of each of the 10 samples using the DNeasy Plant Mini kit (Qiagen, Valencia, CA, USA) and PCR using ITS1 region primers (F: 5′ TCCGTAGGTGAACCTGCGG 3′; R: 5′ AGCGGCGGGTGCTGTTGCAG 3′) was conducted as described previously [14]. The presence or absence of the expected 150 bp fragment was then visualized using a 1% agarose gel.

### Physical wounding experiments

Rather than receive an induction inoculation, some moss gametophores were wounded by piercing with a sterile needle at one end of the explant. Wounded moss gametophores were subjected to a challenge inoculation distal to the site of wounding. The challenge inoculation was carried out as described above and the health of the gametophore was observed for 10 d after challenge. Control gametophores were not wounded prior to challenge. Three replicates containing four samples each, representing a total of twelve gametophores, were performed, and percent survival was recorded.

### β-1,3 glucan experiments

Rather than receiving an induction inoculation or physical wound, some moss gametophores were treated with 3 µl of a 0.5 mg/ml suspension of β-1,3 glucan in glass-distilled water (Sigma-Aldrich, Saint Louis, Missouri, USA). Twenty-four hours after treatment, the moss gametophores were subjected to a challenge inoculation distal to the site of β-1,3 glucan application. The challenge inoculation was carried out as described above, and the health of the gametophores was observed for 10 d after challenge. Control gametophores were treated with 3 µl of water 24 h prior to challenge. Three replicates containing four samples each, representing a total of twelve gametophores, were performed and percent survival was recorded.

### Statistics

Tukey's HSD (Honestly Significant Difference) test was used to determine significant difference among treatment groups in the SAR investigations. T-Tests were used to determine significant difference among treatment groups in the physical wounding and β-glucan experiments. An alpha level of 0.05 was used for statistical significance in all analyses.

## Results

### Progression of *P. irregulare* infection of *A. serpens*

**Figure 2:**
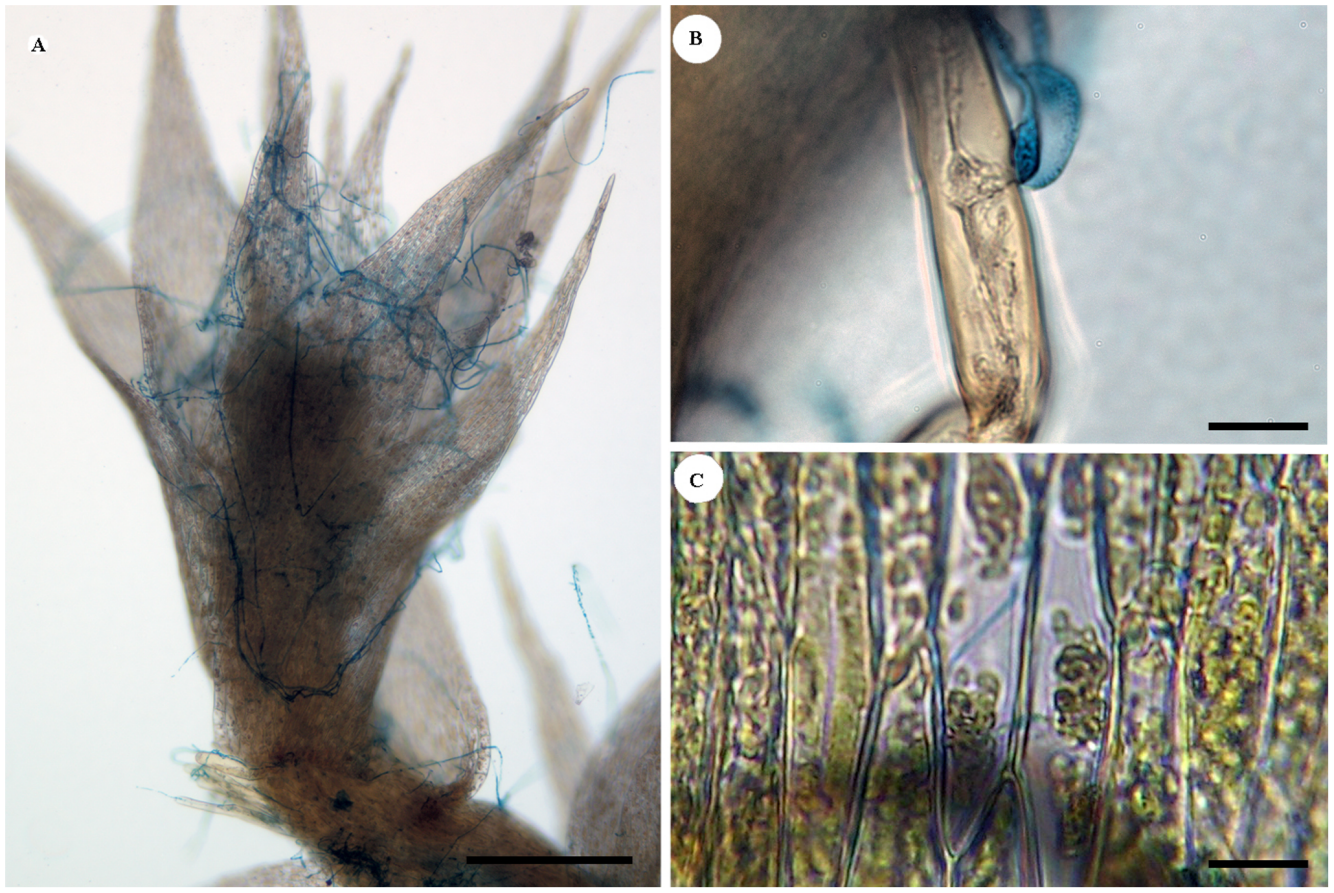
Infection of *A. serpens* by *P. irregulare*. (**A**) Growth of *P. irregulare* (blue) on *A. serpens* (Scale bar = 250 µm), (**B**) formation of appressorium (Scale bar = 50 µm), and (**C**) cytoplasmic shrinkage of host cells 24 h post-inoculation (Scale bar = 50 µm). Fungal cells were stained with trypan blue prior to visualization.

**Figure 3:**
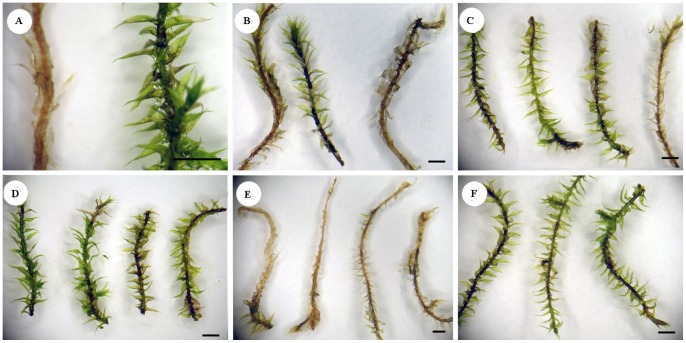
Representative *A. serpens* gametophores following inoculation with *P. irregulare*. (**A**) *A. serpens* gametophores showing complete chlorosis and necrosis (left) and an uninfected control (right) at 10 d after inoculation. (**B**) Three *A. serpens* gametophores that were challenged 10 h after induction. (**C**) Four *A. serpens* gametophores that were challenged 22 h after induction. (**D**) Four *A. serpens* gametophores that were challenged 34 h after induction. (**E**) Four *A. serpens* gametophores that were subjected to physical wounding 24 h prior to challenge. (**F**) Three *A. serpens* gametophores treated on one end with β-1,3 glucan 24 h prior to challenge of the distal end. Gametophores shown in **B**–**F** were observed 7 d after the challenge inoculation. Scale bars = 1 mm.

Microscopic examination of *A. serpens* exposed to *P. irregulare* showed classic infection symptoms (Figure 2), including formation of appressoria with subsequent injection of fungal material (Figure 2B) as well as cytoplasmic shrinkage (Figure 2C). Infected gametophores appeared chlorotic and necrotic with wilted leaves within 10 d of inoculation. Control gametophores retained their initial green color, and leaves remained turgid (Figure 3A, right). Of the moss gametophores receiving no induction inoculation prior to challenge, 85.4% (n = 18) appeared chlorotic and necrotic with wilted leaves (Figure 3A, left); the remaining 14.6% eluded infection and remained green and turgid.

### Critical timing of post-induction manipulation of *A. serpens*

**Figure 4:**
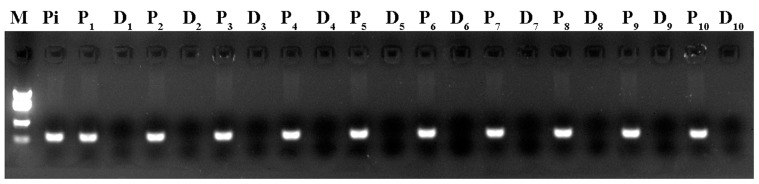
PCR detection of *P. irregulare* on the proximal and distal ends of moss 10 h after induction inoculation. Ten moss gametophores were inoculated with *P. irregulare* at one end. Ten hours after inoculation, the gametophores were cut in half and moved to separate media for an additional 24 h. DNA was then extracted and PCR amplification was attempted using primers that were previously designed for the *P. irregulare* ribosomal ITS1 region [14]. The expected 150 bp fragment was detected in the DNA extracted from *P. irregulare* (Pi) and the proximal ends of all 10 gametophores (P_1_–P_10_) but was not detected in any of the distal ends (D_1_–D_10_).

The Petri dish system we employed ensures that the challenge site receives no prior direct exposure to pathogen. However, sufficient time was necessary to allow the putative signal to move past the cut site to induce changes in the distal end at the eventual challenge site. Pathogen growth could be seen on the distal ends of *A. serpens* samples cut 12 h post induction, indicating that the challenge site had been exposed. Most samples cut 8 h post-induction showed no systemic resistance upon challenge inoculation. Upon visual inspection, the ideal timing of the cut to exclude the pathogen itself while allowing movement of the putative signal was 10 h post-induction. PCR amplification of the *P. irregulare* ribosomal ITS region confirmed that the distal ends of gametophores inoculated 10 h prior to cutting were free of *P. irregulare* (Figure 4).

### Primary inoculation with *P. irregulare* triggers SAR

**Figure 5:**
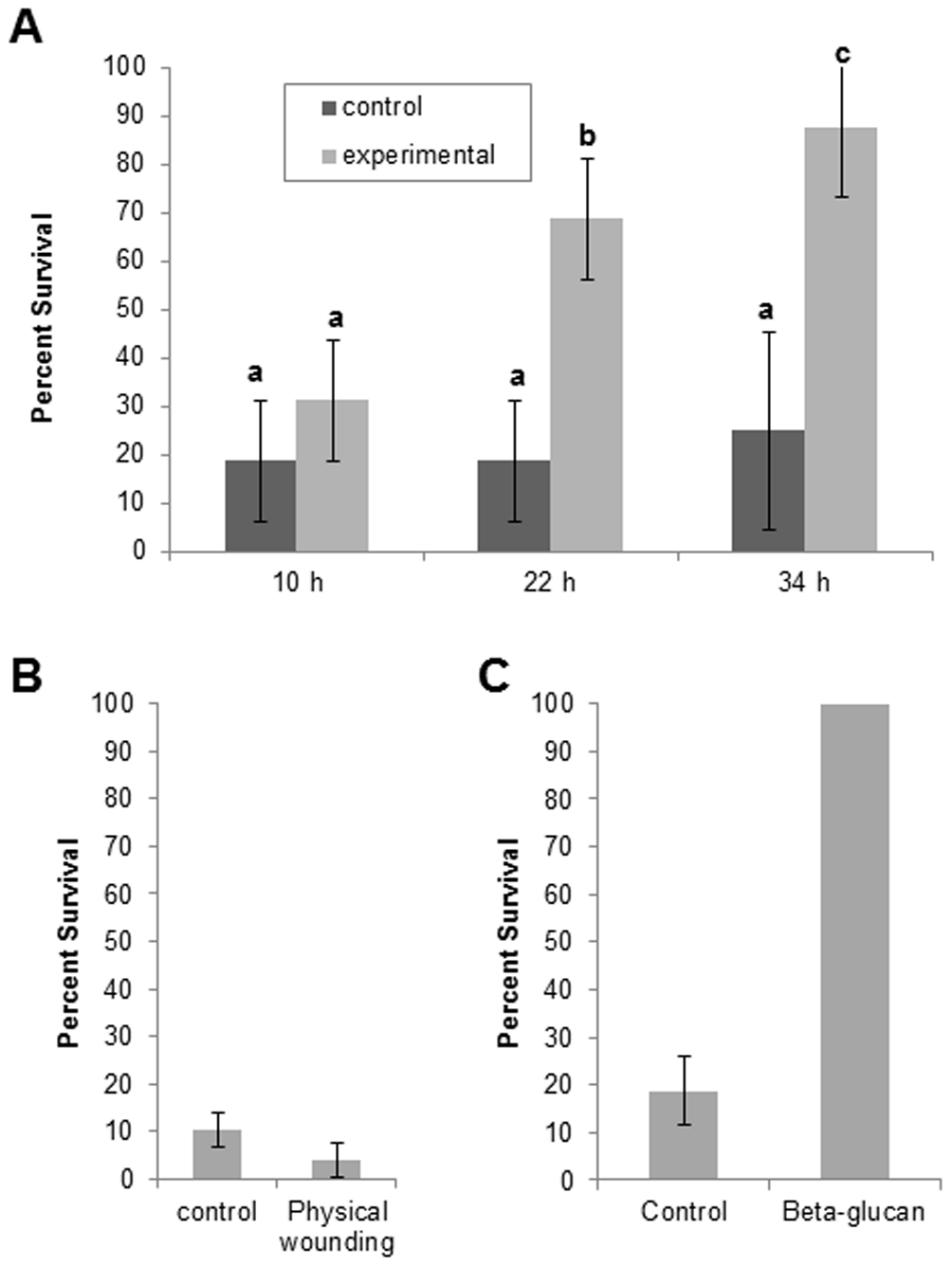
Percent survival of moss gametophores following challenge inoculation with *P. irregulare*. (**A**) Replicates of four moss gametophores were either given a control induction inoculation (no *P. irregulare*) or experimental induction inoculation (*P. irregulare*). Ten hours after the induction inoculation, gametophores were cut, and the distal end of the gametophore was moved to fresh media. The challenge inoculation with *P. irregulare* occurred either 10 h, 22 h, or 34 h after the primary inoculation. 10 d after challenge inoculation, the survival of each of the four gametophores in each sample was recorded. The mean percent survival is shown for each treatment, with standard deviations indicated. Mean percent survival of gametophores was analyzed using the Tukey HSD test. Matching letters above the bars indicates groups that are not significantly different from one another. In **B** and **C**, no induction inoculation was performed. (**B**) Percent survival of moss gametophores 10 d after challenge inoculation after either no pretreatment (control) or 24 h after physical wounding. **C**. Percent survival of moss gametophores 10 d after challenge inoculation after either no pretreatment (control) or 24 h after β-glucan pretreatment. t-Tests were conducted to show in **B** there was no significant difference between wounded and unwounded gametophores (P = 0.10) and in **C** there was a significant difference between β-glucan treated and untreated gametophores (P = 3.69E-05). Treatments were either replicated 4 times (**A**) or 3 times (**B & C**).

Challenge 10 h after induction inoculation (0 h after cutting) yielded a low percentage of survival (31.3%; Figure 5A), and most samples looked phenotypically similar to control samples that did not receive an induction inoculation (Figure 3B). Moss gametophores challenged 34 h after induction inoculation (24 h after cutting) showed a high percentage of survival (87.5%; Figure 5A), and the surviving plants appeared phenotypically similar to uninfected control plants (Figure 3D vs. Figure 3A, right). Twenty-two hours after the induction inoculation (12 h after cutting), the reaction of challenged moss samples was variable as indicated by an intermediate survival rate (68.75%; Figure 5A), and the survivors displayed varying levels of necrosis and chlorosis (Figure 3C). Most plants that survived the challenge inoculation showed some degree of stem darkening. Analysis of the percent survival in these different treatments using the Tukey HSD Test indicates that at the 0.05 level, samples receiving either no induction inoculation or receiving a challenge inoculation 10 h after induction showed similar percent survival. Those samples that were challenged 22 h or 34 h after induction had significantly higher mean percent survival (Figure 5A).

### Wounding does not trigger SAR

Moss gametophores (n = 12) wounded 24 h prior to challenge were phenotypically indistinguishable from unwounded control samples; nearly all of the gametophores became chlorotic and necrotic within 10 d of challenge with *P. irregulare* (Figure 3E, Figure 5B). A two sample t-Test showed no difference in percent survival 10 d after challenge inoculation between unwounded and wounded samples (t = 2.12, d.f. = 4, P = 0.10). Wounded, unchallenged gametophores remained green and appeared similar to healthy control samples (data not shown).

### Treatment with β-1,3 glucan induces SAR

All gametophores (n = 12) treated with β-1,3 glucan 24 h prior to challenge showed neither chlorosis nor necrosis 10 d after challenge (Figure 3F, Figure 5C). A two sample t-Test indicated very significant difference in percent survival 10 d after challenge inoculation between untreated and treated samples (t = 20.0, d.f. = 4, P = 3.69E-05). The stems showed variable levels of darkening 10 d after challenge.

## Discussion

In this study, we devised a novel culture system (Figure 1) and demonstrated the presence of a SAR-like response in a non-vascular plant. The key feature of our approach was the ability to separate in space the primary induction inoculation and secondary challenge inoculation sites on the plants, which allowed the challenge to occur in tissue that was never in direct contact with the pathogen. *P. irregulare* reliably infected *A. serpens*, producing necrosis characteristics for this pathogen. This culture system will prove beneficial for future characterization of the SAR mechanism in moss.

The timing of the cut and transfer between the induction and challenge inoculations was critical: if the tissue was cut too early, the putative SAR signal was not transmitted to the distal end of the moss, whereas if the tissue was cut too late, rapidly growing *P. irregulare* moved to the distal end of the moss, confounding conclusions about the existence of SAR. The optimal time for this cut was determined to be 10 h after induction. All challenged gametophores were visually inspected for the presence of *P. irregulare* on the distal end. In addition, the lack of PCR amplification of the *P. irregulare* ITS1 region on the distal end of inoculated gametophores indicate that any changes in resistance in the distal end were due to the transmission of a signal rather than direct exposure to the pathogen.

Results from 10 h (0 h after cutting) challenge experiments showed SAR was not established within the first 10 h after primary exposure to the pathogen (Figure 5). However, moss plants challenged 34 h after induction inoculation (24 h after cutting) were more resistant to infection by *P. irregulare*. The darkening of the main stem seen in the experimental samples may be the result of an accumulation of phenolic compounds as part of the defense reaction [11], [15], [16]. In order to better understand the timing of SAR, we looked at a time point between 10 and 34 h after induction inoculation. At 22 h after the induction inoculation (12 h after cutting), the reaction of challenged moss samples was variable, as evidenced by an intermediate survival rate (Figure 5). SAR was established in more than half of the moss samples by 22 h post-induction, but 34 h post-induction provided the additional time necessary to ensure the highest level of systemic protection in our experiments.

It is important to note that plants did not achieve 100% protection against plant death at 34 h post-induction, suggesting that the putative SAR signal was not transmitted past the cut point within 10 h of induction in all samples. Lack of signal transmission would prevent the distal ends from undergoing appropriate changes in PR gene expression, rendering the distal ends susceptible to challenge. Possible explanations for observed variability may include differences in the viability and initial health of both the moss samples and pathogen. Despite the variability, the trends strongly support the occurrence of SAR in this non-vascular plant.

Physical wounding experiments did not induce SAR in *A. serpens*, a finding consistent with data showing that physical wounding is incapable of inducing SAR in vascular plants [17]. Therefore, we conclude that rather than simply responding to a physical wound, the moss must be able to sense some specific feature of the pathogen itself. In addition, results of the wounding experiments suggest that simply cutting the moss plants, as part of our method, would not be sufficient to cause induction of the SAR by itself.

The β-1,3 glucan experiments demonstrate that this common oomycete cell wall component can act as a defense elicitor and induce a nearly identical increase in resistance when compared with induction by the pathogen itself. The similar response by moss to both β-1,3 glucan and *P. irregulare* itself suggests that this cell wall component may be detected by the plant during infection by *P. irregulare*. Previous studies implicate the involvement of β-1,3 glucans in SAR in vascular plants [18]. Our results suggest that the moss may possess components of a conserved sensory mechanism for oomycete cell wall material similar to that found in the vascular plants.

**Figure 6:**
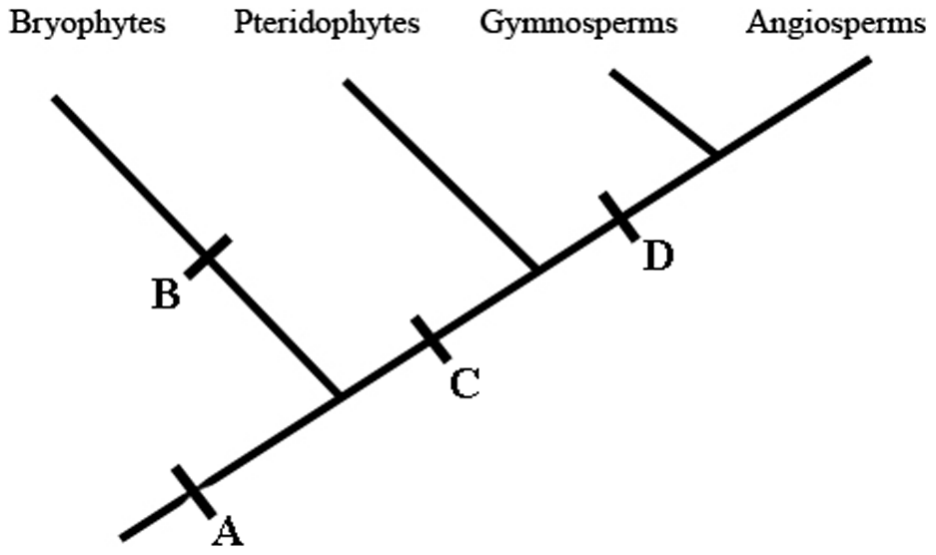
Alternative models for the evolutionary history of SAR in plants. Given the previous reports of SAR in Angiosperms and Gymnosperms, and the current report of an SAR-like mechanism in Bryophytes, three alternate models for the evolutionary history of SAR exist. In one model, SAR predates the divergence of vascular and non-vascular plants (point A in the figure). In this model, the SAR mechanism in all plants should be highly conserved unless SAR mechanisms became divergent, and more specialized for vascular tissue, subsequent to the evolution of vascular plants. Alternately, SAR arose independently in non-vascular plants (point ‘B’ on figure) and in vascular plants (points ‘C’ or ‘D’). In these models, the SAR mechanisms should be less similar between the two groups. Without documentation of SAR in the pteridophytes, it is unknown if SAR in vascular plants arose before pteridophytes diverged from other vascular plants (point ‘C’ on figure) or after this divergence (point ‘D’ on figure).

While our results collectively indicate the existence of a SAR-like mechanism in *A. serpens*, it remains to be seen whether this SAR mechanism, and its components, are conserved relative to those in vascular plants. Three possible evolutionary histories exist for this defense response (Figure 6). One possibility is that SAR arose prior to the divergence of non-vascular and vascular plants, approximately 470 million yr ago [19]. In this scenario, much of the molecular machinery for SAR should be conserved in nearly all land plants. This evolutionary model is supported by findings that both moss and vascular plants have several conserved genes as well as two plant hormones, SA and JA [8], [9], [11]. Alternately, SAR might have evolved independently in non-vascular and vascular plants, either before or after the divergence of the pteridophytes. More work is necessary to confirm its presence or absence in pteridophytes and to examine the degree of similarity between the SAR mechanisms of all types of plants.

Now that a reliable moss-pathogen culture system has been developed and the existence of SAR has been documented in a non-vascular plant, it will be necessary to identify genes in *A. serpens* that are orthologous to PR genes and other SAR-associated genes in vascular plants. Researchers previously detected the induction of four plant defense genes (*CHS*, *PAL*, *LOX*, and *PR-1*) in *P. patens* in response to a pathogen [11]. However, the expression of these genes, and additional PR genes, must be studied to determine if they are induced throughout the plant or just at the site of infection. In addition, the roles of SA, JA, and ethylene in non-vascular SAR should be clarified. Such studies will make it possible to draw more definitive conclusions regarding when the SAR mechanism arose in plants. It is also necessary to determine if pteridophytes possess a SAR mechanism and to further characterize SAR in gymnosperms to determine the degree of conservation of this process in all plant lineages.

Another remaining question pertains to the identity of the signal and how it is dispersed throughout the moss plant. Even in vascular plants, the identity of the SAR signal remains elusive; however, most well-supported candidates, including methyl salicylate and lipid-based signals, are likely transported through the phloem [20]. Additional volatile signals, such as ethylene or methyl jasmonate, which is known to play a role in increasing resistance to herbivory [21], could also play a signaling role in SAR [22]. The possibility that multiple signals, including both phloem-mobile and volatile, are used in SAR by vascular plants has been discussed [20], [23]. Mosses lack a well- developed vascular system; however, transmittance of the signal may occur through the primitive transport system in moss made up of water-conducting cells (hydroids) and sugar-conducting cells (leptoids) [24]. In this model, it is possible that the same phloem-mobile signal molecule(s) from vascular plants are used. Alternately, it is possible that mosses use a volatile SAR signal only, and the existence of phloem-mobile signals in vascular plants evolved after the divergence of vascular and non-vascular plants. Finally, the signal could be passed from cell to cell through simple diffusion or via pathways involving Rac/Rop GTPase [25], calcium ions [26], or cellular redox changes [27].

In summary, our study is the first to demonstrate the existence of SAR in non-vascular plants, and we describe a reliable model for future studies of SAR in moss. The novel culture system we have developed should also be very useful in future studies for elucidating the evolutionary history of plant defense systems. Such future studies are necessary to reveal similarities and differences with SAR in vascular plants to help elucidate the evolutionary history of this important defense mechanism.
